# Generation of a recombinant antibody for sensitive detection of *Pseudomonas aeruginosa*

**DOI:** 10.1186/s12896-022-00751-9

**Published:** 2022-08-04

**Authors:** Gyu-Min Lim, Joo-Kyung Kim, Eun-Jung Kim, Chang-Soo Lee, Wooseong Kim, Byung-Gee Kim, Hee-Jin Jeong

**Affiliations:** 1 Interdisciplinary Program in Bioengineering, Seoul National University, Seoul, 08826 Republic of Korea; 2 BioMAX/N-Bio Institute, Institute of Bioengineering, Seoul National University, Seoul, 08826 Republic of Korea; 3 Department of Chemical Engineering and Applied Chemistry, Chungnam National University, Daejeon, 34134 Republic of Korea; 4 College of Pharmacy and Graduate School of Pharmaceutical Sciences, Ewha Womans University, Seoul, 03760 Republic of Korea; 5 Department of Biological and Chemical Engineering, Hongik University, Sejong, 30016 Republic of Korea

**Keywords:** *Pseudomonas aeruginosa*, Recombinant antibody, Enzyme-linked immunosorbent assay, HEK293F cells, Point-of-care testing

## Abstract

*Pseudomonas aeruginosa* (*P. aeruginosa*) is a major pathogen that causes nosocomial infections and often exhibits antibiotic resistance. Therefore, the development of an accurate method for detecting *P. aeruginosa* is required to control *P. aeruginosa*-related outbreaks. In this study, we established an enzyme-linked immunosorbent assay method for the sensitive detection of three *P. aeruginosa* strains, UCBPP PA14, ATCC 27853, and multidrug-resistant ATCC BAA-2108. We produced a recombinant antibody (rAb) against *P. aeruginosa* V‐antigen (PcrV), which is a needle tip protein of the type III secretion system of *P. aeruginosa* using mammalian cells with high yield and purity, and confirmed its *P. aeruginosa* binding efficiency. The rAb was paired with commercial anti-*P. aeruginosa* Ab for a sandwich ELISA, resulting in an antigen-concentration-dependent response with a limit of detection value of 230 CFU/mL. These results suggest that the rAb produced herein can be used for the sensitive detection of *P. aeruginosa* with a wide range of applications in clinical diagnosis and point-of-care testing.

**Supplementary Information:**

The online version contains supplementary material available at 10.1186/s12896-022-00751-9.

## Introduction

*Pseudomonas aeruginosa* (*P. aeruginosa*) is a Gram-negative opportunistic pathogen that causes infections and conditions such as cystic fibrosis, lung transplant, cancer, and AIDs predispose patients to *P. aeruginosa* infection [1–5]. Eradication of *P. aeruginosa*-related infectious diseases is difficult, as *P. aeruginosa* acquires resistance to antibiotics [6]. Therefore, early detection of *P. aeruginosa* is critical. Conventional *P. aeruginosa* detection methods are based on the evaluation of the growth activity of bacteria under optimized culture conditions or antimicrobial susceptibility testing [7–10]. However, these bacterial culture-based methods require 3–4 days to identify *P. aeruginosa* because the culture procedure for extending the number of cells up to the detectable scale requires several days. Moreover, these results are sometimes inconsistent owing to sample contamination during culture. Thus, these traditional approaches need to be improved to achieve fast and accurate detection in clinical practice.

Recently, automated systems with simple operating procedures have been developed to reduce turnaround times for identifying microbial species [11–13]. However, these systems provide low accuracy and sensitivity for the identification of *P. aeruginosa*. Nucleic acid-based assays using polymerase chain reaction (PCR) have been evaluated for detection of *P. aeruginosa* and several *P. aeruginosa*-specific genes, such as exotoxin A genes, have been discovered for the PCR assays [9, 14, 15]. The PCR-based *P. aeruginosa* detection method showed higher sensitivity and shorter detection time than culture-based methods [15]. However, the specificity of PCR-based method is sometimes associated with false results. Mass spectrometry (MS) analysis has been applied to the identification of *P. aeruginosa* as an efficient microbial identification technology by mapping bacterial proteins because MS analysis provides rapid, accurate, sensitive, and high-throughput detectability [16, 17]. However, MS analysis requires costly equipment, and a highly specialized technician is needed to interpret the complex data.

The enzyme-linked immunosorbent assay (ELISA), which utilizes the highly specific binding between antibody (Ab) and antigen, has been applied to quantify *P. aeruginosa*. Ueda et al. detected antibodies against *P. aeruginosa* in horse serum using ELISA [18]. Granstrom et al. developed an ELISA system for the detection of antibodies against exotoxin A or phospholipase C from patients infected with *P. aeruginosa* [19]. Fomsgaard et al. used an anti-lipopolysaccharide antibody to detect *P. aeruginosa* [20]. Dogru et al. performed ELISA by targeting exotoxin A, elastase, and alkaline protease to detect early *P. aeruginosa* infection in patients [21]. However, as those assays target *P. aeruginosa*-secreting molecules, there is the possibility of false-positive results. Therefore, it is necessary to develop a more precise method for direct detection of *P. aeruginosa*.

The pathogenesis of Gram-negative bacteria is associated with bacterial toxicity by the type III secretion system (T3SS), which is present on the surface of bacteria and passages bacterial effectors into infected cells [22]. *P. aeruginosa* V-antigen, PcrV, is a protein on the needle of T3SS and contacts host cells to transport multiple virulence factors into host cells [23]. As PcrV is surface accessible and serotype independent, anti-PcrV Ab has been shown to prevent T3SS-mediated cytotoxicity [24, 25]. MEDI3902, a bispecific monoclonal Ab against both PcrV and the polysaccharide synthesis locus (Psl) exopolysaccharide, which is expressed in HEK293 cells, is under clinical evaluation [26]. Tabor et al. generated a Fab-type MEDI3902 by papain digestion of human IgG1, V2L2MD, which was derived from recombinant PcrV-immunized mice. They confirmed that the Fab bound to the PcrV expressed on the cell surface of *P. aeruginosa* respiratory strains, including multiple antibiotic-resistant strains [27]. However, although Fab can be used for further applications, it is generated by enzyme cleavage, which is expensive to obtain. Moreover, enzymatic cleavage of Abs can cause destruction of its antigen-binding domain and/or the fragment itself [28]. Moreover, Fab has a half-life of 12–20 h in human serum, whereas full-sized Abs have a longer half-life (approximately three weeks) [29, 30]. To this end, we aimed to generate a full-sized recombinant Ab (rAb) against PcrV and to describe its application as an ELISA reagent for detecting *P. aeruginosa*. In this study, we generated a novel rAb that could directly detect *P. aeruginosa* and confirmed its sensitivity. We performed a sandwich ELISA by pairing the rAb with a commercial Ab and confirmed that the selected pair could detect the clinical level of *P. aeruginosa*.

## Results and Discussion

### Construction of recombinant antibody expression gene

**Fig. 1:**
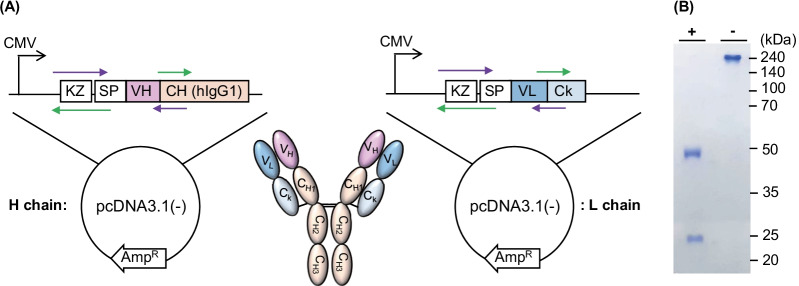
**A** Schematic images of full-sized antibody (middle) and the plasmid DNA maps for the expression of H chain (left) and L chain (right) of recombinant antibody. KZ, SP, VH, CH, VL, and Ck indicates kozak sequence, signal peptide, variable heavy chain, constant heavy chain, variable light chain, and constant kappa light chain. Nucleotide sequence of a kozak sequence and signal peptide for H chain and L chain was 5’-gccaccatgggatacagaatgcagctgctgagctgtatcgccctgtctctggccctggtcaccaattct-3’ and 5’-gccaccatgggatacagaatgcagctgctgagctgtatcgccctgtctctggccctggtcaccaatagc-3’, respectively. **B** SDS-PAGE analysis of recombinant antibody. “ + ” indicates a sample reduced by adding DTT and heating, and “-” indicates a nonreduced sample

We genetically synthesized codon-optimized VH and VL genes of MEDI3902 [26] and introduced each DNA into the pcDNA3.1(-) vector, which is a widely used vector for protein or antibody expression in mammalian cells. Prior to the VH or VL sequence, we added the Kozak sequence followed by the IL-2 signal sequence to increase the expression yield. We prepared each plasmid and used them for transient co-transfections of HEK293F cells (Fig. 1A). MEDI3902 is a bispecific antibody that targets both Psl, which is related to an adhesin, and PcrV that influence an infection. In this study, we focused on PcrV as a target antigen of the antibody because several studies have reported that Psl is located not only on the surface of *P. aeruginosa* but also on the biofilm, which is formed by *P. aeruginosa* [31–33]. Therefore, we were concerned regarding the possibility that the antibody against Psl recognized not only *P. aeruginosa* but also the biofilm formed by multiple strains of *P. aeruginosa*. This could lead to a low selectivity when the anti-Psl Ab is used for detecting *P. aeruginosa* in a real sample, such as a mixture of various pathogen-included clinical samples.

### Expression and purification of recombinant antibody

We expressed rAb through a suspension culture of HEK293F cells, which has been widely used for large-scale production of proteins, including antibodies. We injected two plasmids, pcDNA3.1::anti-*P. aeruginosa* H chain and pcDNA3.1::anti-*P. aeruginosa* L chain, with polyethylenimine and cultured the cells. The supernatant was collected and purified using protein A (PA) affinity beads, and the buffer was changed to PBS using ultrafiltration. Next, we performed SDS-PAGE analysis with or without reduction to resolve the structure of the generated rAb (Fig. 1B, Additional file 1: Fig. S1). After denaturing the sample using DTT and heating, two bands were observed, which were corresponded to the H chain and L chain, whose amino acid-based calculated size was 51.9 kDa and 25.7 kDa, respectively. In the case of the native sample without denaturation, no band around 51.9 kDa or 25.7 kDa was observed, indicating that excess H and L chains were not present. Only the expected full-sized Ab was present in the sample, and the rAb was successfully expressed with correct folding. It is worth noting that almost no extra bands were observed in the sample, indicating that the purification was very high only after PA affinity purification without additional size-exclusion chromatography, which can cause the loss of Ab. We confirmed that 3.36 mg of purified rAb was obtained per 150 mL of culture.

### Antigen-binding efficiency of recombinant antibody

**Fig. 2:**
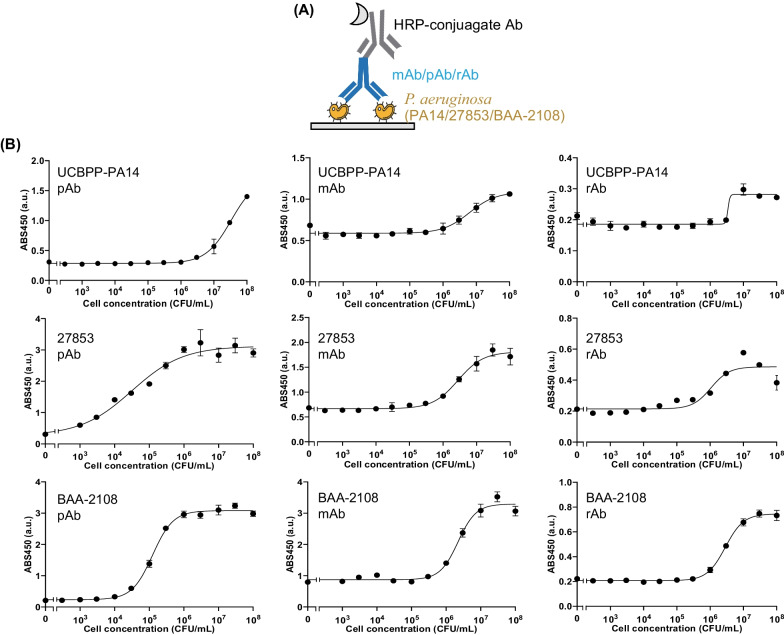
Confirmation of the antigen-binding efficiencies of commercial and recombinant Abs against ATCC 27853, UCBPP-PA14, and ATCC BAA-2108 strains. **A** schematic representation of the indirect ELISA, **B** ELISA signals of Abs against each strain. Error bars represent ± 1 SD (n = 3)

We confirmed the antigen-binding efficiency of rAb against three *P. aeruginosa* strains: *P. aeruginosa* UCBPP-PA14, ATCC 27853, and ATCC BAA-2108. *P. aeruginosa* UCBPP-PA14 is a susceptible strain, isolated from a human burn patient [34]. ATCC 27853 is a susceptible strain, isolated from a hospital blood specimen [35]. BAA-2108 is a multidrug-resistant *P. aeruginosa* strain that was isolated from a cystic fibrosis patient during a clinical test for evaluating the efficacy of aerosolized tobramycin [36]. We cultured the three strains and diluted them. We plated the cells on a 96-well plate and performed indirect ELISA using rAb as the primary antibody. At that time, we investigated the antigen-binding efficiency of both rAb and two commercial anti-*P. aeruginosa* Abs, monoclonal Ab (mAb) and polyclonal Ab (pAb). We used HRP-conjugated goat anti-human IgG-Fc antibody, HRP-conjugated goat anti-mouse IgG2a antibody, and HRP-conjugated goat anti-rabbit IgG antibody as a secondary antibody for rAb, mAb, and pAb, respectively (Fig. 2A). As a result, the signals of total nine ELISA platforms with different antibodies and antigens increased in an antigen-concentration dependent manner, indicating the antigen-binding efficiency of each antibody against each strain (Fig. 2B and Table 1). When we compared the responses of three antibodies against *P. aeruginosa* UCBPP-PA14, two commercial Abs showed a broader detection range than rAb, whereas the half maximal effective concentration (EC50) values were similar. When we used ATCC 27853 as an antigen, pAb showed a very low limit of detection (LOD) value (35 CFU/mL). As the pAb was generated by immunizing whole cells of the ATCC 27853 strain, the result that the pAb showed higher activity against ATCC 27853 than against the other two strains was consistent with our hypothesis and supported the usefulness of this Ab for sensitive detection of ATCC 27853. In the case of ATCC BAA-2108, the LOD values of mAb and rAb were similar and the LOD value of pAb was higher than that of the other two Abs. Overall, among the three Abs, which showed antigen-binding efficiency, commercial pAb showed a higher response than mAb and rAb. Nonetheless, the sensitivities of these Abs against each antigen, except pAb against ATCC 27853, were not sufficient to detect clinical level; 1–10^4^ CFU/mL [37].

**Table 1:** Half maximal effective concentration (EC50) and limit of detection (LOD) values of antibodies that were determined from the titration curves of indirect ELISA. n.d. = not detected

*P. aeruginosa* strain	Antibody	EC50 (CFU/mL)	LOD (CFU/mL)
UCBPP-PA14	Polyclonal antibody (pAb)	3.08 ± 0.31 × 10^7^	2.92 × 10^6^
UCBPP-PA14	Monoclonal antibody (mAb)	7.46 ± 3.27 × 10^6^	4.69 × 10^6^
UCBPP-PA14	Recombinant antibody (rAb)	n.d	n.d
ATCC 27,853	pAb	5.03 ± 0.98 × 10^4^	3.50 × 10^2^
ATCC 27,853	mAb	2.77 ± 0.37 × 10^6^	4.01 × 10^5^
ATCC 27,853	rAb	1.01 ± 0.28 × 10^6^	2.43 × 10^5^
ATCC BAA-2108	pAb	1.30 ± 0.08 × 10^5^	4.42 × 10^5^
ATCC BAA-2108	mAb	2.15 ± 0.27 × 10^6^	1.37 × 10^5^
ATCC BAA-2108	rAb	2.91 ± 0.30 × 10^6^	7.62 × 10^5^

### Sandwich ELISA for sensitive detection of *P. aeruginosa*

In the case of indirect ELISA, the directions of antigen molecules for attachment to the plate differ because of the random seeding of the antigen molecules to the plate. Thus, the epitope of the antigen can be attached to the plate. In sandwich ELISA, two antibodies, capturing antibody and detecting antibody, bind specifically to each epitope. Therefore, the binding capability between antigen and antibody in sandwich ELISA is higher than that in indirect ELISA, resulting in higher sensitivity. Moreover, the selectivity of sandwich ELISA is usually higher than that of indirect ELISA because two antibodies are used to “sandwich” the antigen, which has an advantage when complex samples are used, because only the antigen is specifically immobilized to the capturing antibody rather than the entire complexed sample to the plate. Thus, sandwich ELISA can be more versatile when used for detecting pathogens in complicated in vivo samples, such as food and blood. In addition, *P. aeruginosa* can form a biofilm when attached to the surface of a 96-well plate for indirect ELISA [38]. Therefore, sandwich ELISA is more suitable than indirect ELISA for forming planktonic cells.

**Fig. 3:**
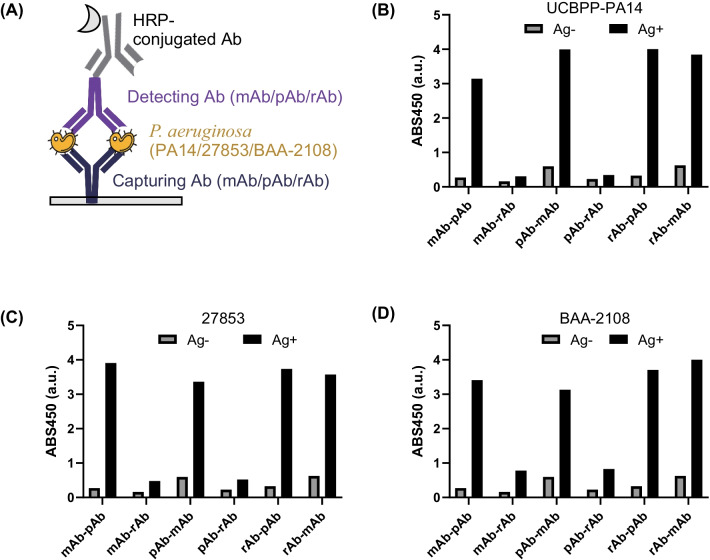
**A** Schematic representation of sandwich ELISA. **B** ELISA signals of each Ab pair with 10^8^ CFU/mL of UCBPP-PA14, **C** ELISA signals of each Ab pair with 10^8^ CFU/mL of 27853, and **D** ELISA signals of each Ab pair with 10^8^ CFU/mL of ATCC BAA-2108. Captions below x-axis of each bar graph represent capturing Ab-detecting Ab

Based on these points, we performed indirect ELISA as well as sandwich ELISA. The most important step in sandwich ELISA is selecting the best pair for capturing and detecting Abs. Therefore, we first screened pairs from the combinations of pAb, mAb, and rAb. Each Ab was seeded onto a plate, and the wells were blocked. Afterwards, we added 10^8^ CFU/mL of UCBPP-PA14, ATCC 27853, ATCC BAA-2108 or PBS and washed the wells. Next, we added a capturing Ab, followed by an HRP-conjugated Ab, which binds to each capturing Ab (Fig. 3A). All pairs showed higher signals in the presence of antigen than in the absence of antigen (Fig. 3B–D). Among them, four pairs, mAb–pAb, pAb–mAb, rAb–pAb, and rAb–mAb (in the order of capturing Ab–detecting Ab), showed a relatively higher signal to background ratio (S/B) than the other two pairs, mAb–rAb and pAb–rAb. Although the responses of mAb–rAb and pAb–rAb can be improved by optimizing the ELISA conditions and/or by using more appropriate HRP-conjugated Ab that has higher secondary antibody-binding affinity, we moved to the next step by using these four pairs because these selected pairs showed high enough responses with S/B against three pathogens.

**Fig. 4:**
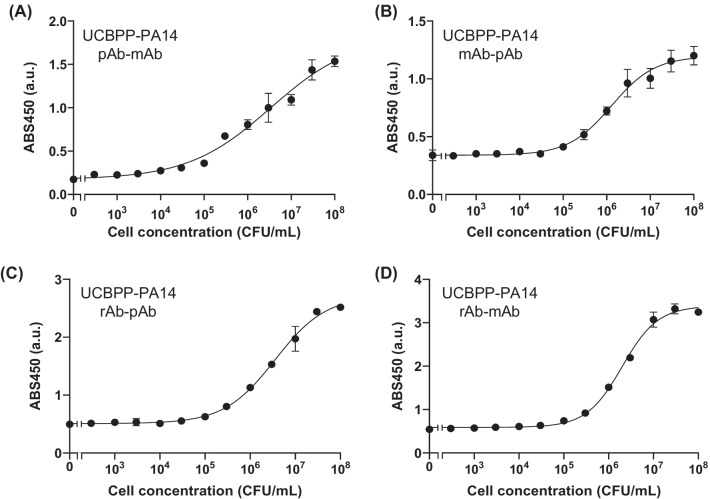
ELISA signals of **A** polyclonal Ab-monoclonal Ab pair, **B** monoclonal Ab-polyclonal Ab pair, **C** recombinant Ab-polyclonal Ab pair, and **D** recombinant Ab-monoclonal Ab pair with UCBPP-PA14. Error bars represent ± 1 SD (n = 3)

**Fig. 5:**
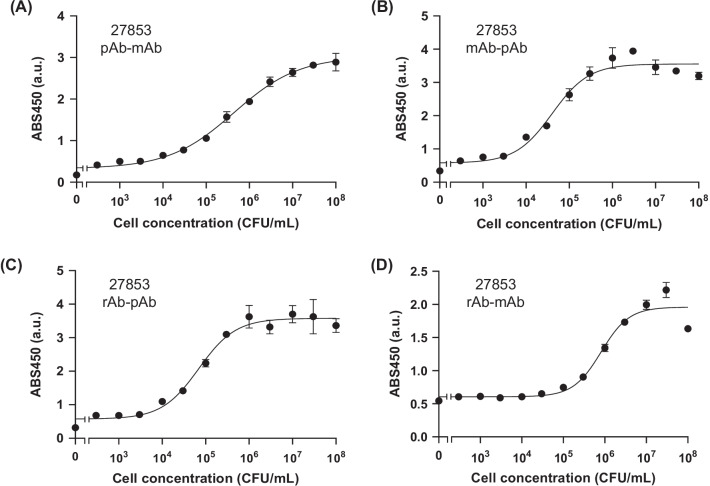
ELISA signals of **A** polyclonal Ab-monoclonal Ab pair, **B** monoclonal Ab-polyclonal Ab pair, **C** recombinant Ab-polyclonal Ab pair, and **D** recombinant Ab-monoclonal Ab pair with ATCC 27853. Error bars represent ± 1 SD (n = 3)

**Fig. 6:**
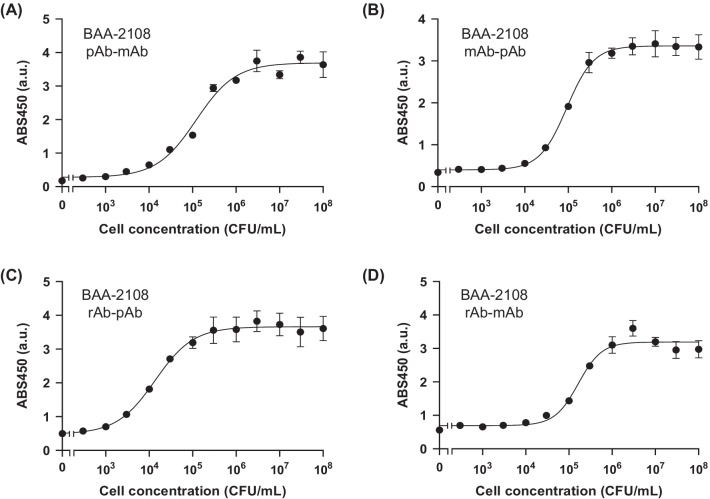
ELISA signals of **A** polyclonal Ab-monoclonal Ab pair, **B** monoclonal Ab-polyclonal Ab pair, **C** recombinant Ab-polyclonal Ab pair, and **D** recombinant Ab-monoclonal Ab pair with BAA-2108. Error bars represent ± 1 SD (n = 3)

We seeded various concentrations of antigen on the plate and performed a sandwich ELISA. Antigen-concentration-dependent responses were observed with the use of all antibody pairs against each antigen, and EC50 and LOD values were calculated (Table 2). When we used UCBPP-PA14 as an antigen, the EC50 values between pairs were similar (approximately 10^6^ CFU/mL), but the pAb-mAb pair showed the lowest LOD value of 85 CFU/mL, which was significantly lower than others (10^4^–10^5^ CFU/mL) (Fig. 4). In the case of ATCC 27853, the mAb-pAb pair showed the lowest EC50 (4.10 ± 0.39 × 10^4^ CFU/mL) and LOD (2.04 × 10^3^ CFU/mL) (Fig. 5). For ATCC BAA-2108, the rAb-pAb pair showed a lower EC50 (1.39 ± 0.22 × 10^4^ CFU/mL) and LOD (230 CFU/mL) than the others (Fig. 6). It is worth noting that all LOD values using an rAb for detecting three strains of *P. aeruginosa* were below 10^4^ CFU/mL, indicating its usefulness for the sensitive detection of each pathogen.

**Table 2:** Half maximal effective concentration (EC50) and limit of detection (LOD) values of antibodies that were determined from the titration curves of sandwich ELISA

Antigen	Capturing antibody	Detecting antibody	EC50 (CFU/mL)	LOD (CFU/mL)
UCBPP-PA14	Monoclonal antibody (mAb)	Polyclonal antibody (pAb)	1.43 ± 0.44 × 10^6^	1.39 × 10^5^
UCBPP-PA14	pAb	mAb	4.62 ± 2.80 × 10^6^	8.50 × 10^2^
UCBPP-PA14	Recombinant antibody (rAb)	pAb	4.10 ± 1.68 × 10^6^	3.34 × 10^4^
UCBPP-PA14	rAb	mAb	2.00 ± 0.06 × 10^6^	7.46 × 10^4^
ATCC 27,853	mAb	pAb	4.10 ± 0.39 × 10^4^	2.04 × 10^3^
ATCC 27,853	pAb	mAb	4.56 ± 1.11 × 10^5^	5.78 × 10^3^
ATCC 27,853	rAb	pAb	7.01 ± 0.79 × 10^4^	7.85 × 10^3^
ATCC 27,853	rAb	mAb	8.21 ± 0.95 × 10^5^	6.30 × 10^4^
ATCC BAA-2108	mAb	pAb	9.19 ± 0.56 × 10^4^	3.79 × 10^3^
ATCC BAA-2108	pAb	mAb	1.20 ± 0.04 × 10^5^	7.70 × 10^2^
ATCC BAA-2108	rAb	pAb	1.39 ± 0.22 × 10^4^	2.30 × 10^2^
ATCC BAA-2108	rAb	mAb	1.63 ± 0.17 × 10^5^	1.84 × 10^4^

## Conclusion

In this study, we generated a recombinant anti-PcrV Ab using HEK293F cells and confirmed its binding efficiency to three strains of *P. aeruginosa*. We used the rAb with commercial Ab for sandwich ELISA, resulting in high sensitivity for detecting three pathogens. In particular, when we used the rAb-pAb pair to detect multidrug-resistant ATCC BAA-2108, the LOD value was very low (230 CFU/mL). In the case of UCBPP-PA14 and ATCC 27853, the pairs of rAb and commercial Ab showed a low LOD on the order of 10^4^ CFU/mL, even though the value was higher than that of the pairs of commercial Abs, 10^3^–10^4^ CFU/mL. As the rAb can be produced with high yield and purity by using the method described in this study, it has a high potential for being used for practical detection at a low cost, while commercial Abs are expensive. Moreover, as the information including DNA sequence and a production method of rAb was opened in this study, rAb can be manipulated to obtain further sensitivity and for conjugating functional molecules such as peptides, enzymes, and fluorescent dyes.

It is worth mentioning that it takes 19–20 h to perform the ELISA, which we presented herein. This is in contrast to the conventional *P. aeruginosa* detection methods, such as bacterial culture-based methods, which take 3–4 days. Moreover, since the ELISA described in this study can detect a small amount of *P. aeruginosa* without pre-culture of the pathogen, it is distinguishable from the traditional methods that have relatively low sensitivity and thus require pre-incubation of the pathogens to acquire enough cells for detection. Recently, Cepheid Inc. (Sunnyvale, California, USA) developed a powerful automated real-time PCR-based *P. aeruginosa* diagnostic system, which could detect various *P. aeruginosa* strains, including PA-CoI, NCTC 13437, 758, B92A, CoI 1, 73999, MKAM, 70,450–1, 5344, 3985, 4032, 3424, and 92, at an LOD of 50–10^6^ CFU/mL within only 48 min [39]. However, the three strains used in this study, UCBPP PA14, ATCC 27853, and ATCC BAA-2108, were not addressed by this system.

Considering these advantages, this rapid and sensitive *P. aeruginosa* detection method using recombinant antibodies can minimize sample consumption and save cost; thus, it can be widely applied for efficient and practical detection of *P. aeruginosa*.

## Methods

### Materials

The Herculase II Fusion DNA polymerase was obtained from Agilent (Seoul, Korea). Oligonucleotides were obtained from Bionics (Seoul, Korea). Infusion kit was obtained from Takara (Seoul, Korea) Plasmid maxiprep kit was obtained from GeneAll Biotechnology (Seoul, Korea). Protein A beads were obtained from GE healthcare (Piscataway, NJ, USA), Disposable gravity column were obtained from Bio-Rad (Daejeon, Korea). Ultrafiltration devices were obtained from Millipore (MWCO 3 k; Seoul, Korea). 96 well ELISA plate was obtained from SPL (Seoul, Korea). HEK293F cell line (FreeStyle293-F cells) was obtained from Thermo (Waltham, Massachusetts, USA). Freestyle293 media and HT supplement were obtained from Gibco (Waltham, MA, USA). FBS was obtained from Afronteir (Seoul, Korea). PEI was obtained from Polysciences (Warrington, Pennsylvania, USA). Goat Anti-mouse IgG HRP and PA agarose resin were obtained from Pierce (Seoul, Korea). Mouse anti-*P. aeruginosa* monoclonal IgG2a [B11] that was generated by immunizing outer membrane protein of *P. aeruginosa* was obtained from Abcam (Cat. No. PA1-73,116, Cambridge, UK). Rabbit anti-*P. aeruginosa* polyclonal IgG that was generated by immunizing whole cells of *P. aeruginosa* ATCC 27853 strain was obtained from Thermo (Cat. No. PA1-73116, Waltham, Massachusetts, USA). HRP-conjugated goat anti-rabbit IgG (H + L), and HRP-conjugated goat anti-mouse IgG2a were obtained from Invitrogen (Waltham, Massachusetts, USA). HRP-conjugated goat anti-human IgG-Fc antibody was from Sino Biological (Beijing, China). Other chemicals and reagents, unless otherwise indicated, were from Sigma.

### Gene construction of recombinant anti-P. aeruginosa antibody

DNA was amplified by polymerase chain reaction (PCR) using Herculase II Fusion DNA polymerase according to the manufacturer’s protocol. The signal sequence, followed by VH of anti-PcrV Fab expressing gene (PDB: 6CYF) [27], was amplified using primers IL2H-fuF (5’-cgccaccatgggatacagaatgcagctgctg-3’) and 6CYF_VH_fuR (5’-ttgtagaggcgctagacactgtcactgtggtgcc-3’), and synthesized DNA (Table 3) as template DNA. The PCR product was ligated to a vector DNA, which was amplified by PCR using primers IL2H-fuR (5’-ctcagcagctgcattctgtatcccatggtggcgg-3’) and 6CYF_VH-fuF (5’-accacagtgacagtgtctagcgcctctacaaagg-3’), and pcDNA3.1::anti-MMP9 H chain [40] as a template DNA, using the Infusion enzyme, resulting in pcDNA3.1::anti-*P. aeruginosa* H chain. The signal sequence followed by VL of anti-PcrV Fab-expressing gene was amplified using primers, IL2L-fuF (5’-gccaccatgggatacagaatgcagctgctgag-3’) and 6CYF_VL_fuR (5’-ggccactgttctcttgatttccaccttggtgcc-3’), and synthesized DNA (Table 3) as template DNA. The PCR product was ligated to a vector DNA, which was amplified using primers IL2L-fuR (5’-cagcagctgcattctgtatcccatggtggcgg-3’) and 6CYF_VL-fuF (5’-ccaaggtggaaatcaagagaacagtggccgctcc-3’), and pcDNA3.1::anti-MMP9 L chain [40] as a template DNA, using the Infusion enzyme, resulting in pcDNA3.1::anti-*P. aeruginosa* L chain. Large-scale plasmid preparation was performed using the plasmid maxiprep system.

**Table 3:** Sequences of variable domains in recombinant antibody

	VH	VL
Nucleotide	gaggtgcagctggtggaatctggcggcggacttgttcaacctggcggctctctgagactgagctgtgccgcttccggcttcacctttagcagctacgccatggactgggtccgacaggctcctggcaaaggccttgaatgggtgtccgccatcaccatgtctggcatcaccgcctactacaccgacgacgtgaagggcagattcaccatcagccgggacaacagcaagaacaccctgtacctgcagatgaacagc	gccatccagatgacacagagccccagcagcctgtctgcctctgtgggagacagagtgaccatcacctgtagagccagccagggcatcagaaacgacctcggctggtatcagcagaagcctggcaaggcccctaagctgctgatctacagcgccagcacactgcagagcggagtgcctagcagattttctggcagcggctccggcaccgatttcaccctgaccatatctagcctgcagcctgaggacttcgccacc
Amino acid	EVQLVESGGGLVQPGGSLRLSCAASGFTFSSYAMDWVRQAPGKGLEWVSAITMSGITAYYTDDVKGRFTISRDNSKNTLYLQMNSLRAEDTAVYYCAKEEFLPGTHYFYGMDVWGQGTTVTV	AIQMTQSPSSLSASVGDRVTITCRASQGIRNDLGWYQQKPGKAPKLLIYSASTLQSGVPSRFSGSGSGTDFTLTISSLQPEDFATYYCLQDYNYPWTFGQGTKVEI

### Production of recombinant anti-*P. aeruginosa* antibody

Mammalian cell-based recombinant antibody expression was performed according to our previous research [41] with slight modifications. HEK293F cells were maintained in 50 mL growth medium (Gibco Freestyle 293) in a 250 mL flask in an atmosphere of 8.0% CO2 and at 37 °C. After suspension cell culture, 3 × 10^5^–3 × 10^6^ cells/mL in the supernatant were obtained, and the subcultured cells were transferred to 200 mL media at a density of 1 × 10^6^ cells/mL. Then, 1.25 μg/mL pcDNA3.1::anti-*P. aeruginosa* H chain and pcDNA3.1::anti-*P. aeruginosa* L chain genes of the antibody were co-transfected into HEK293F cells using 7.5 μg/mL of PEI according to the manufacturer’s protocol. After 7 days of incubation at 120 rpm at 8.0% CO_2_ at 37 °C, the supernatant was transferred to a 50 mL tube, and the cells were collected by centrifugation. rAbs obtained from each cell supernatant were purified using PA agarose beads according to the manufacturer’s protocol as follows: 10 μL of PA beads was added to 1 mL of cell supernatant and incubated at RT for 1 h. After washing three times with 500 μL of PBS, the rAb was eluted using 100 μL of 0.1 M glycine (pH 2.5) and immediately neutralized by adding 10 μL of PBS. The rAb concentration was calculated by measuring the absorbance at 280 nm using a NanoDrop spectrophotometer.

### P. aeruginosa culture

The cells of three *P. aeruginosa* strains, UCBPP PA14 [34], ATCC 27853 [35], and ATCC BAA-2108 [36], were cultured in LB media by growing cultures overnight to log phase at 37 °C at 200 rpm. The cells at OD600 = 0.5 were washed three times with PBS and diluted with the same buffer.

### Indirect ELISA

The antigen-binding activities of Abs were tested using indirect ELISA as follows: 100 μL of serially diluted *P. aeruginosa* was immobilized on a 96-well plate for 16 h at 4 °C. The wells were then filled with 300 μL of PBSB (PBS containing 3% bovine serum albumin (BSA)) for 1 h at 25 °C, and then washed three times with PBSB. Subsequently, 300 ng of antibody in 100 μL (3 ng/μL) of PBSB was added and incubated for 1 h at 25 °C. After washing three times with 300 μL of PBSB, the bound protein was probed with 100 μL of 0.03 ng/μL HRP-conjugated antibody in PBSB for 1 h at 25 °C. The wells were washed three times with 300 μL of PBSB and developed with 50 μL of tetramethylbenzide (TMB) solution. After incubation for 10 min, the reaction was stopped with 50 μL of 1 N H2SO4. The absorbance at 450 nm was measured using a microplate reader (Thermo Lab Systems). Dose − response curves were generated by fitting the absorbance values using Graphpad Prism software. The EC50 value was calculated using a 4-parameter logistic equation of the software. The LOD value was calculated based on the following equation: LOD = mean blank + 1.645 × SD_blank_ + 1.645 × SD_low concentration sample_ [42].

### Sandwich ELISA

300 ng of Ab in 100 μL PBS was immobilized on a 96-well plate for 16 h at 4 °C. The wells were then filled with 300 μL of PBSB for 1 h at 25 °C, and then washed three times with 300 μL of PBSB. Subsequently, 100 μL of *P. aeruginosa* was added and incubated for 1 h at 25 °C. After washing three times with 300 μL of PBSB, 300 ng rAb or commercial Ab was added and incubated for 1 h at 25 °C. After washing three times with 300 μL of PBSB, the bound antibody was probed with 100 μL of 0.03 ng/μL HRP-conjugated goat anti-mouse IgG antibody for mAb, or 100 μL of 0.03 ng/μL HRP-conjugated goat anti-rabbit IgG antibody for pAb, or 10,000-times diluted HRP-conjugated goat anti-human IgG antiody for rAb in PBSB for 1 h at 25 °C. The wells were washed three times with 300 μL of PBSB and developed with 50 μL of TMB solution. After incubation for 15 min, the reaction was stopped with 50 μL of 1 N H2SO4. The absorbance at 450 nm was measured using a microplate reader. Dose − response curves were generated by fitting the absorbance values using Graphpad Prism software. The EC50 value was calculated using a 4-parameter logistic equation of the software. The LOD value was calculated based on the following equation: LOD = mean blank + 1.645 × SD_blank_ + 1.645 × SD_low concentration sample_ [42].

## Supplementary Information

**Additional file 1: Fig. S1.** Complete original electrophoresis gel.

## Data Availability

All data generated or analyzed during this study are included in this published article.
