# Supplementary material for: Generation of a recombinant antibody for sensitive detection of Pseudomonas aeruginosa
Source: BMC Biotechnol. 2022 Aug 4;22:21. doi: 10.1186/s12896-022-00751-9 (PMC9354424; doi:10.1186/s12896-022-00751-9)
Supplement: Supplementary file 1 — Additional file 1: Fig. S1. Complete original electrophoresis gel. [file 12896_2022_751_MOESM1_ESM.docx]

Legend for the Supplementary Figure 1

Supplementary Figure 1: Complete original electrophoresis gel. Marker: commercially available protein ladder (DokDo-MARK, Cat. No. EBM-1032), + : recombinant *P. aeruginosa* antibody, which was dissolved in reducing buffer (625 mM Tris (pH 6.8), 10% glycerol, 2% SDS, 0.01% bromophenol blue, 100 mM dithiothreitol) and loaded to the gel after heating at 95℃ for 10 min. – : recombinant *P. aeruginosa* antibody, which was dissolved in non-reducing buffer (625 mM Tris (pH 6.8), 10% glycerol, 2% SDS, 0.01% bromophenol blue) and loaded to the gel without heating. * : samples of another experiment [1], ** : bovine serum albumin.

[1] Kim J-K, Lim G-M, Kim E-J, Kim W, Lee C-S, Kim B-G, Jeong H-J: Generation of Recombinant Antibodies in Hek293f Cells for the Detection of Staphylococcus Aureus. *ACS Omega* **2022**, 7(11):9690-9700.
